# Mechanisms and Efficacy of Traditional Chinese Medicine in Heart Failure

**DOI:** 10.3389/fphar.2022.810587

**Published:** 2022-02-24

**Authors:** Anzhu Wang, Wei Zhao, Kaituo Yan, Pingping Huang, Hongwei Zhang, Zhibo Zhang, Dawu Zhang, Xiaochang Ma

**Affiliations:** ^1^ Graduate School, China Academy of Chinese Medical Sciences, Beijing, China; ^2^ Xiyuan Hospital, China Academy of Chinese Medical Sciences, Beijing, China; ^3^ Yidu Central Hospital of Weifang, Weifang, China; ^4^ Xiyuan Hospital, Beijing University of Chinese Medicine, Beijing, China; ^5^ National Clinical Research Center for Chinese Medicine Cardiology, Beijing, China

**Keywords:** heart failure, traditional Chinese medicine, microvascular circulation, myocardial energy metabolism, inflammation, oxidative stress

## Abstract

Heart failure (HF) is one of the main public health problems at present. Although some breakthroughs have been made in the treatment of HF, the mortality rate remains very high. However, we should also pay attention to improving the quality of life of patients with HF. Traditional Chinese medicine (TCM) has a long history of being used to treat HF. To demonstrate the clinical effects and mechanisms of TCM, we searched published clinical trial studies and basic studies. The search results showed that adjuvant therapy with TCM might benefit patients with HF, and its mechanism may be related to microvascular circulation, myocardial energy metabolism, oxidative stress, and inflammation.

## Introduction

Heart failure (HF) is a clinical syndrome characterized by with dyspnea, fatigue, and hydroncus, which is caused by decreased cardiac output or elevated endocardial pressure resulting from a change in the cardiac structure or function (McDonagh et al., 2021). As shown in the 2017 Global Burden of Disease Study, the number of patients with HF is about 64.34 million, which makes HF an epidemic public health problem (Disease et al., 2018). The morbidity of HF in adults from developed countries is between 1 and 2% (Groenewegen et al., 2020). Data from the USA in 2019 showed that 6.2 million people aged above 20 years old have HF, and HF morbidity is estimated to increase by 46% from 2012 to 2030 (Benjamin et al., 2019). The 2012–2015 China Hypertension Survey found that the morbidity of HF in adults aged 35 years and over was 1.3% (Wang Z et al., 2018). Moreover, the incidence of heart failure increases with age. Data from the Heart Failure Association of the European Society indicates that 1-year all-cause mortality of acute heart failure (AHF) and chronic heart failure (CHF) are 23.6 and 6.4%, respectively (Crespo-Leiro et al., 2016). The INTERnational Congestive Heart Failure (INTER-CHF) prospective cohort study showed that HF 1-year all-cause mortality was 16.5%, which in Africa was 34%, in Southeast Asia was 15%, in China was 7%, in South America was 9%, and in the Middle East was 9% (Dokainish et al., 2017).

In clinical practice, HF can be divided into HF with reduced ejection fraction [HFrEF, left ventricular ejection fraction (LVEF) ≤ 40%] and HF with preserved ejection fraction (HFpEF, LVEF ≥ 50%) based on the LVEF (Bozkurt et al., 2021). Research into the treatment of HFrEF has formed a new quadruple chemotherapy consisting of angiotensin-converting enzyme inhibitors, beta-blockers, mineralocorticoid receptor antagonists, and sodium-dependent glucose transporters 2 inhibitors in medication (McMurray et al., 2014; McMurray et al., 2019; Packer et al., 2020). Although these treatments show prognostic benefits, the 5-years mortality after hospitalization of patients with HFrEF is still high, even up to 75.3% (Shah et al., 2017). Among all research aimed at HFpEF published to date, only the EMPEROR-Preserved Trial proved that medicine could improve prognosis (Anker et al., 2021). Previous guidelines defined HF (LVEF = 41–49%) as HF with a mid-range ejection fraction (Ponikowski et al., 2016). Meanwhile, the latest European Society of Cardiology guidelines defined it as HF with mildly reduced ejection fraction (HFmrEF); however, we lack prospective experimental evidence for patients with HFmrEF. All analyses and suggestions are based on post hoc analyses of HFrEF and HFpEF (McDonagh et al., 2021). Therefore, the treatment of HF still faces great challenges. One the one hand, we need to focus on improving life qualities of patients with HF, in addition to its notable morbidity and mortality (Fiuzat et al., 2020). On the other hand, medicine exploitation needs new therapeutic targets, such as microvascular circulation, myocardial energy metabolism, inflammation, and mitochondrial function (Reddy et al., 2020; Ghionzoli et al., 2021).

Traditional Chinese Medicine (TCM), based on its own theory, has resulted in Chinese medicinal herbs being used widely in the therapy of HF. For example, many anti-HF prescriptions are recorded in textbooks (such as Zhenwu decoction, Shengmai powder, and Lingguizhugan decoction), while only a few of them have been studied strictly (Fu et al., 2010). Chinese medicinal herbs have been used for a long time to cure heart failure. By contrast, research into the effects and mechanisms of TCM are still in the initial stage. In this article, we summarize recent development in this field, with the aim of clarifying the mechanisms of Chinese medicinal herbs in therapy for HF and to provide new directions for the development and clinical application of HF therapeutic drugs.

## Clinical Studies of Traditional Chinese Medicine in Treating Heart Failure

Considering the large number of studies about TCM treatment in HF, we only searched two databases, the Chinese Clinical Trial Registry (http://www.chictr.org.cn) and ClinicalTrials.gov (http://www.clinicaltrials.gov), and then obtained relevant data from the China National Knowledge Infrastructure (https://www.cnki.net/) and PubMed (https://pubmed.ncbi.nlm.nih.gov/), which resulted in 13 studies being selected (Table 1). The search date was August 28, 2021. Two investigators (Anzhu Wang and Pingping Huang) screened the literature separately, and a third investigator (Xiao-Chang Ma) checked the search results. Two investigators (Anzhu Wang and Pingping Huang) used the Cochrane risk of bias assessment tool to assess the risk of bias for the included studies. Disagreements in the process were resolved through discussion and consultation with a third investigator (Xiaochang Ma). The search process and quality assessment were placed in Supplementary Figures S1, S2.

**TABLE 1:** Clinical studies of TCM to treating HF.

Study	Registration number	N	Patient cohort	TCM intervention measures	Length of study	Primary endpoints	Secondary endpoints
Li et al. (2013)	ChiCTR-TRC-11001478	512	LVEF ≤ 40%; NT-proBNP ≥450 pg/ml; NYHA II-IV	Qili Qiangxin capsules: *Astragulus embranaceus (Fisch.) Bge., Panax ginseng C. A. Mey., Aconitum carmichaelii Debx., Salvia miltiorrhiza Bge., Lepidium apetalum Willd, Alisma orientalis* (*Sam.*)*Juzep, Polygonatum odoratum (Mill.) Druce, Cinnamomum cassia Presl, Carthamus tinctorius L., Periploca sepium Bge., and Citrus reticulata Blanco*	12 Weeks	NT-proBNP	CCEs, NYHA, LVEF, LVED, 6MWD, MLHFQ
Mao et al. (2020)	NCT01555320	640	Ischemic heart disease; LVEF ≤45% or a history of HF/related clinical symptoms for more than 3 months; NYHA II-IV	Qishen Yiqi dripping pills: *Astragulus embranaceus (Fisch.) Bge., Salvia miltiorrhiza Bge., Panax notoginseng (Burk.) F. H. Chen, Dalbergia odorifera T.Chen*	6 Months	6MWD	Composite endpoints, BNP, LVEF, NYHA, MLHFQ
Xian et al. (2016)	ChiCTR-TRC-12003063	240	CHF combined with coronary artery disease; Qi-Yin deficiency; NYHA II-IV	Shenmai Injection: *Panax ginseng C.A.Mey., OPhiopogon japonicus (L.f) Ker-Gawl*	7 Days	NYHA	6MWD, SF-36 hearth survey score, TCM syndrome score, LVEF, BNP
Wang et al. (2020)	ChiCTR1800016293	120	Unstable condition requiring further treatment in the hospital; NYHA II–IV	Shenmai Injection: *Panax ginseng C.A.Mey*., *OPhiopogon japonicus (L.f) Ker-Gawl*	7 Days	FFAs, Glucose, LA, PA, BCAAs	NYHA, TCM syndrome score, LVEF, LVIDd, LVIDs, BNP
Wang et al. (2019)	ChiCTR-TRC-12002857	160	Coronary heart disease; CHF during acute aggravation; Yang and Qi deficiency; LVEF ≤50%; NYHA III-IV	Shenfu Injection: *Panax ginseng C.A. Mey., Aconitum carmichaelii Debx*	7 Days	NYHA, TCM syndrome score	Lee’s CHF score, 6MWD, LVEF, The incidence rate of cardiovascular events and HF emergency/rehospitalization
Xu et al. (2011)	ChiCTR-TRC-08000257	118	CHF during acute aggravation Qi deficiency LVEF ≤40%; NYHA III-IV	Huangqi Injection: *Astragulus embranaceus* (*Fisch.*)*Bge*	7 Days	LVEF	Dyspnea situatio, NYHA, Clinical sign, Tei index
Wang et al. (2017b)	ChiCTR-TRC-12002061	465	CHF caused by ischemic heart disease or dilated cardiomyopathy; 35% ≤ LVEF ≤50%; 720/24 h ≤ VPCs ≤ 10,000/24 h; NYHA II-III	Shensong Yangxin capsules: *Astragulus embranaceus (Fisch.) Bge., OPhiopogon japonicus (L.f) Ker-Gawl., Cornus officinalis Sieb. et Zucc., Salvia miltiorrhiza Bge., Ziziphus jujuba Mill. var. spinosa (Bunge) Hu ex H.F.Chou, Taxillus chinensis(DC.) Danser, Paeonia lactiflora Pall.; Paeonia veitchii*) *Lynch, Eupolypha gasinensis Walk, Naradostachys jatamansi DC., Coptis chinensis Franch., Schisandra sphenantheraRehd.et Wils., Fossilia Ossia Mastodi*	12 Weeks	VPCs	LVEF, LVEDD, NT-proBNP, NYHA, 6MWD, MLHFQ.
Xian et al. (2015)	ChiCTR-TRC-09000549	228	NYHA II-Ⅲ and Stage C of HF; Qi-Yin deficiency, blood stasis and water stagnation	Yangxinkang Tablets: *Panax ginseng C.A.Mey., Astragulus embranaceus* (*Fisch*.) *Bge., OPhiopogon japonicus* (*L.f*) *Ker-Gawl., Schisandra chinensis* (*Turcz*.) *Baill., Ilex pubescens Hook. et Arn., Leonurus japonicus Houtt., Lepidium apetalum Willd*	4 Weeks	NYHA, CM syndromes, Symptom score, Sign Score, MLHFQ	General characteristics
Li X. X et al. (2021)	ChiCTR1900022036	76	NYHA Ⅱ-Ⅲ and stage C of HF; Qi deficiency, blood stasis and water stagnation	Qishen Taohong Granule: *Astragulus embranaceus* (*Fisch.*) *Bge, Codonopsis pilosula* (*Frnnch*.) *Nannf, Salvia miltiorrhiza Bge, Prunus persica* (*L*.) *Batsch, Carthamus tinctorius L., Morus allba L., Lepidium apetalum Willd, Polyporus umbellatus* (*Pers*) *Fries, Lycopur Lucidus Turcz. var. hirtus Regel*	4 Weeks	NYHA, LVEF, CHFQLS	6MWD, CM syndrome score, Symptom score, Sign score, NT-proBNP.
Tang, (2020)	ChiCTR-INR-17010696	108	Insufficient heart blood and heart Yang; LVEF <40%	Yangxinxue granule: *Bupleurum chinense DC, Ostrea gigas Thunberg, Ziziphus jujuba Mill. var. spinosa* (*Bunge*) *Hu ex H.F. Chou, Angelica sinensis* (*Oliv.*) *Diels, Rehmannia glutinosa Libosc, Paeonia lactiflora Pall., Acanthopanax gracilistylus W.W. Smith, Ligusticum chuanxiong Hort., Panax ginseng C. A. Mey., Polygonatum odoratum* (*Mill*.) *Druce, Cinnamomum cassia Presl*	6 Months	1-Year cardiovascular-related mortality	BNP, Length of hospital stay, Number of hospitalization, Rehospitalization frequency and length, 6MWD, MLHFQ
						Recurrence frequency of AHF	
Wang et al. (2017b)	ChiCTR-TRC- 08000059	340	CHF admitted to hospital; LVEF ≤50%; NYHA Ⅱ-Ⅳ	Traditional Chinese Medicine preparations (Shenfu injection, Shenmai injection, Danhong injection, Qili Qiangxin Capsules, Buyiqiangxin tablets): The composition of Buyiqiangxin tablets is as follows: *Panax ginseng C. A. Mey., Astragulus embranaceus (Fisch.) Bge., Periploca sepium Bge., Salvia miltiorrhiza Bge, OPhiopogon japonicus (L.f) Ker-Gawl., Lepidium apetalum Willd*	1 ± 2 Weeks and 6 Months	All-cause mortality	BNP, 6MWD, LVEF, MLHFQ
Liu, (2020)	ChiCTR2000030921	60	HFpEF; NYHA Ⅰ-Ⅲ	Yangyin Shuxin prescription: *Cornus officinalis Sieb. et Zucc., OPhiopogon japonicus (L.f) Ker-Gawl., Poygonautun kingianum Coll. et Hemsl*., *Coptis chinensis Franch, Trionyx sinensis Wiegmann, Salvia miltiorrhiza Bge., pheretima aspergillum (E.perrier), Pinellia ternata (Thunb.) Breit., Trichosanthes kirilowii Maxim., Citrus aurantium L*	14 ± 3 Days	Cardiac ultrasound index (,two-dimensional ultrasound,pulsed-wave doppler, tissue doppler imaging, speckle tracking imaging), Exercise tolerance (Peak VO₂, minute ventilation/VCO₂ slope)	TCM syndrome score, MLHFQ, BNP
Gao et al. (2017)	NCT01939236	64	Coronary heart disease; NYHA II-III; LVEF <40%	Chinese herbal medicine granules: *Astragulus embranaceus (Fisch.) Bge, Codonopsis pilosula (Frnnch.) Nannf., Salvia miltiorrhiza Bge, Prunus persica (L.) Batsch, Carthamus tinctorius L., Paeonia veitchii Lynch*	28 Days	Metabolomics analysis	6MWT, LVEF

6MWD (6-min walking distance), BCAAs (Branched-chain amino acids), BNP (Brain natriuretic peptide), CCEs (Composite cardiac events), CHF (Chronic heart failure), CHFQLS (Quality of Life measured by the CHF Integrated Chinese and Western Medicine Survival Scale), CM (Chinese medicine), ECG (Electrocardiograph), FFAs (Free fatty acids), HF (Heart Failure), LA (Lactic acid), LVED (Left ventricular end-diastolic diameter), LVEDD (Left ventricular end diastolic diameter), LVEF (Left ventricular ejection fraction), LVIDd (Left ventricular internal diastolic diameter), LVIDs (Left ventricular internal dimension systole), MLHFQ (Minnesota Living with Heart Failure Questionnaire), NT-proBNP(N-terminal pro-B-type natriuretic peptide), NYHA (New York Heart Association), PA (Pyroracemic acid), SF-36 (Short-form 36), TCM (Traditional Chinese Medicine), VPCs (Ventricular premature complexes).

### Qili Qiangxin Capsules

In 2013, Li et al. (2013) reported a multicenter, randomized, double-blind, parallel-group, placebo-controlled study on the efficacy and safety of Qili Qiangxin capsules in 512 patients with CHF. After 12 weeks of treatment, the level of N-terminal pro-B-type natriuretic peptide (NT-proBNP) in the two groups was significantly lower than baseline. However, reduction in the Qili Qiangxin capsules group was markedly larger than that in placebo group [240.15 pg/ml (−23.15, 1113.85) vs. 0.00 pg/ml (−286.00, 800.00), *p* = 0.002]. The therapeutic effects of Qili Qiangxin capsules were clearly better than that of the placebo (*p* < 0.05) in terms of composite cardiac events, New York Heart Association (NYHA) functional classification, LVEF, 6-min walking distance (6MWD), and Quality of life assessment using the Minnesota Living with Heart Failure Questionnaire (MLHFQ). In terms of safety, the differences between two groups for serious adverse events (SAEs) and adverse events (AEs) were not statistically significant (*p* < 0.05). Elevated levels of circulating NT-proBNP contribute to the diagnosis of HF and are linked to increased mortality and morbidity in HF patients (McDonagh et al., 2021). The findings revealed that Qili Qiangxin capsules can significantly lower NT-proBNP levels, indicating that patients may have a better prognosis with long-term therapy. To test this theory, a large, randomized, controlled trial with all-cause death as the outcome is required.

### Qishen Yiqi Dripping Pills

In 2020, Mao et al. (2020) reported a prospective randomized, double-blind, multicenter, placebo-controlled study on the efficacy and safety of Qishen Yiqi dripping pills that enrolled 640 patients with ischemic heart failure (IHF). After 6 months of treatment, the level of 6MWD in the two groups was significantly higher than baseline. However, increase in the Qishen Yiqi dripping pills group was obviously greater than that in the placebo group (336.15 ± 100.84 m to 374.47 ± 103.09 m vs. 334.40 ± 100.27 m to 340.71 ± 104.57 m, *p* < 0.001). Compared with those in the placebo group, the MLHFQ grades of Qishen Yiqi dripping pills group were better and the NYHA functional classification was ameliorated (*p* < 0.05). Although Qishen Yiqi dripping pills increased LVEF and BNP during a 6-month period, the effects were not substantially larger than those seen in the placebo group. This was also linked to a lack of meaningful difference in composite clinical events at 6 months and 1 year (follow-up), despite a tendency for decreased HF hospitalizations at 6 months. To thoroughly assess the impact of Qishen Yiqi dripping pills on clinical events in IHF, longer-term medication and follow-up may be required. In terms of safety, AEs in the Qishen Yiqi dripping pills group were less common and milder.

### Shenmai Injection

In 2016, Xian et al. (2016) delivered a randomized, double-blind, multicenter, placebo-controlled study on the efficacy and safety of Shenmai injection in 240 patients with CHF. After 7 days of treatment, the levels of the NYHA functional classification in the two groups were lower than baseline. However, the proportion of improved patients in the Shenmai injection group was significantly bigger than placebo group (NYHA Ⅰ: 22.8 vs. 8.8%, NYHA Ⅱ: 12.3 vs. 14.9%, NYHA Ⅲ: 28.1 vs. 19.3%, and NYHA Ⅳ: 7.0 vs. 4.4%, *p* = 0.001). In 6MWD, the short-form 36 (SF-36) hearth survey score and the TCM syndrome score, Shenmai injection therapy was more effective than the placebo (*p* < 0.05). For safety, treatment with Shenmai injection within 1 week was well tolerated with no apparent safety concerns (*p* > 0.05). It is worth noting that, despite indications of improvements in these endpoints, there is no evidence in this trial to demonstrate any benefits in long-term results of Shenmai injection. If a long-term duration is envisaged, more detailed study should be conducted.

In 2020, Wang et al. (2020) initiated a controlled experiment in which 120 patients with HF requiring further treatment in the hospital were randomly assigned to one of three groups: Shenmai injection, trimetazidine, or placebo. After 7 days of treatment, compared with the control group, Shenmai injection could inhibit the production of free fatty acids (FFAs, 452.88 ± 226.62 vs. 571.42 ± 209.40 μmol/L, *p* < 0.05) and branched-chain amino acids (BCAAs, 0.55 ± 0.17 vs. 0.47 ± 0.17 nmol/μL, *p* < 0.05), improved the NYHA functional classification, and raised the level of TCM score associated brain natriuretic peptide (BNP, *p* < 0.05). The advantage of Shenmai injection in HF may be due to an improvement in energy metabolism that was more noticeable than that seen after trimetazidine therapy. However, the number of serological metabolic indexes was limited, and there were no direct associations between metabolic indexes and enhanced cardiac function, nor were there any links between metabolic indexes. As a result, greater study into changes in serological metabolism after Shenmai injection therapy in HF is needed to corroborate these findings.

### Shenfu Injection

In 2019, Wang et al. (2019) reported a randomized, double-blinded, multicenter, placebo-controlled trial of Shenfu Injection in 160 patients with CHF during the acute phase of symptom aggravation (Yang and Qi Deficiency Syndrome). After 7 days of treatment, compared with the control group, Shenfu Injection could improve the NYHA classification (78.38 vs. 61.43%, *p* = 0.0026), and increase the TCM syndrome score (89.19 vs. 60.00%, *p* < 0.001), Lee’s HF score, and 6MWD (*p* < 0.05). In terms of safety, treatment with Shenfu Injection within 1 week showed no statistical differences in the occurrence of AEs and adverse drug reactions (ADRs) (*p* > 0.05). However, this study was limited to a few places, and the observation duration was brief. Furthermore, we found no significant differences in several endpoint markers, such as composite cardiac events (CCEs) or fatalities, across these groups.

### Huangqi Injection

In 2011, Xu et al. (2011) reported a randomized controlled trial (RCT) of Huangqi injection in 128 patients with acute decompensated CHF(Qi deficiency syndrome). After 7 days of treatment, compared with the control group, Huangqi injection could improve the LVEF (37.98 ± 12.77 vs. 31.06 ± 10.36, *p* = 0.003), strengthen dyspnea, and increase the NYHA functional classification and Tei index (*p* < 0.05). In terms of safety, Huangqi injection was well tolerated, with no AEs. In addition, this trial designed the application dose of Huangqi injection at 40 ml/d, which was double the conventional dosage, suggesting that the application of Huangqi injection for the treatment of AHF can be increased, although fluid intake must be tightly regulated. Currently, there are no thorough pharmacodynamic and pharmacokinetic studies as well as large-scale RCTs on Huangqi injection for the treatment of HF, which need to be examined further.

### Shensong Yangxin Capsules

In 2017, Wang et al. (2017b) reported a randomized, double-blind, multicenter placebo-controlled trial of Shensong Yangxin capsules in 465 patients with CHF with frequent ventricular premature complexes (VPCs). After 12 weeks of treatment, compared with the placebo group, the Shensong Yangxin capsules group had significantly fewer total number of VPCs on a 24-h ambulatory electrocardiogram (1538 ± 2187 vs. 2746 ± 3889, *p* < 0.05). In addition, it also increased NT-proBNP, LVEF, NYHA functional classification, 6MWD, and the MLHFQ score (*p* < 0.05). In terms of safety, the differences between two groups for SAEs were not statistically significant (*p* > 0.05). On the basis of conventional CHF therapy, Shensong Yangxin capsules were shown to have the advantages of reducing VPCs and enhancing cardiac function with good compliance in this research. However, because the frequency of VPCs varies daily or on a regular basis, a longer period of ECG, such as 72 h or even 7 days, is seen to be more compelling. Because the 12-weeks timeframe in this study was so short, a bigger size and longer duration of a well-designed clinical trial should be conducted for a more thorough examination of Shensong Yangxin capsules in CHF patients with VPCs.

### Yangxinkang Tablets

In 2105, Xian et al. (2015) reported a randomized, double-blind, multicenter, placebo-controlled study of Yangxinkang Tablets in 228 patients with CHF. After 4 weeks of treatment, compared with the placebo group, Yangxinkang Tablets could improve the NYHA functional classification, Chinese medicine (CM) syndromes, the symptom score, the Sign Score, and quality of life measured by the MLHFQ score (*p* < 0.05). No prominent ADRs were noted during the study. Yangxinkang tablets were shown to relieve clinical symptoms in CHF patients in this trial, although the lack of laboratory markers made the findings less reliable.

### Qishen Taohong Granules

In 2021, Li X. X et al. (2021) reported a single-center, prospective, randomized, controlled clinical trial of Qishen Taohong granules in 76 patients with CHF. After 4 weeks of treatment, compared with trimetazidine, the Qishen Taohong granules could improve the NYHA classification (74.29 vs. 54.29%, *p* < 0.05), quality of life measured using the CHF Integrated Chinese and Western Medicine Survival Scale (CHFQLS; 13.82 ± 6.04 vs. 7.49 ± 2.28, *p* < 0.05). Treatment with Qishen Taohong granules also showed a superior performance in 6MWT, CM syndrome, shortness of breath, fatigue, gasping, general edema, and NT-proBNP levels (*p* < 0.05). No remarkable AEs was revealed during the study. The findings of this study should be interpreted with care due to the small sample size and short observation period. Furthermore, because this study lacked quantitative markers directly connected to energy metabolism, it was unable to evaluate if improved qi deficiency symptoms equated to improved energy metabolism.

### Yangxinxue Granules

In 2020, Tang (2020) reported a multicenter, randomized, double-blind, placebo-controlled study of Yangxinxue granules in 108 patients with CHF. After 6 months of treatment, as well as 6 months of follow-up, although the differences between two groups for 1-year cardiovascular-related disease mortality were not statistically significant (13.33 vs. 6.52%, *p* > 0.05), the attack rate and rehospitalization rate were much lower for patients with AHF in the Yangxinxue granule group (*p* < 0.05). Moreover, the Yangxinxue granules could reduce the BNP level, and improve the 6MWT and MLHFQ scores (*p* < 0.05). No significant ADRs were noted during the study (*p* > 0.05). In addition to the standard treatment, Yangxinxue granules could significantly reduce the number of episodes of AHF and BNP levels, indicating that Yangxinxue granules had definite clinical efficacy in the treatment of CHF. The study included four research centers, but all were located in China’s Sichuan province and may not reflect regional differences.

### TCM Preparations

In 2017, Wang et al. (2017a) reported a prospective, single-blind, randomized, controlled, and multicenter clinical trial of TCM preparations in 340 patients with CHF. During hospitalization, the control group received polarized solution and the treatment group received Danhong injection with Shenfu injection or Shenmai injection. After discharge, all patients were treated with Qiliqiangxin capsules and Buyiqiangxin tablets or a placebo for 6 months. After 6 months, the Kaplan-Meier curves revealed a significantly improved trend in the treatment group’s cumulative survival rate compared to the control group. Although the treatment group ceased taking TCM and got the same therapy as the control group after 6 months, the treatment group’s cumulative survival rate remained higher than the control group until the 12th month (*p* = 0.208). More research is needed to identify whether this favorable trend is the result of TCM’s delayed effect or of other reasons. There was no significant difference between the groups in BNP, Lee’s HF scores and LVEF (*p* > 0.05). The treatment group showed greater improvement in 6MWT and MLHFQ (*p* < 0.05). No significant ADRs were noted during the study (*p* > 0.05).

### Yangyin Shuxin Prescription

In 2020, Liu (2020) reported a randomized, controlled clinical trial of Yangyin Shuxin prescription in 60 patients with HFpEF. The study evaluated the diastolic function of patients using various methods including two-dimensional ultrasound echocardiography, pulsed-wave doppler, tissue doppler imaging and speckle tracking imaging. After 14 ± 3 days of treatment, the mean difference in untwisting rate was 0.14% (95% CI: 0.07–0.21%, *p* < 0.001) and the mean difference in left atrial volume index was −4.99 ml/m^2^ (95% CI: −7.09 to −2.99 ml/m^2^, *p* < 0.001) between the two groups on the basis of conventional treatment, suggesting that Yangyin Shuxin prescription could improve diastolic function in patients with HFpEF. This research’s diagnostic criteria correspond to various guidelines, but there were issues such as a limited number of patients, a short observation duration, and insufficient persuasive power of outcome indicators, necessitating a more well-designed clinical investigation.

### Chinese Herbal Medicine Granules

In 2017, Gao et al. (2017) reported a randomized, double-blind controlled clinical trial of Chinese herbal medicine granules in 64 patients with HFpEF of Qi deficiency and blood stasis syndrome. After 28 days of treatment, compared with the placebo group, Chinese herbal medicine granules could improve the LVEF and 6MWT (*p* < 0.05). By comparing metabolic profiles, 9 biomarkers, including 2-arachidonoylglycerophosphocholine, lysophosphatidylethanolamine (LPE) 16:0, phosphatidylserine 21:0, LPE 20:4, LPE 18:0, linoleic acid, LPE 18:2, 4-hydroxybenzenesulfonic acid, and LPE 22:6, may be particularly important for the effect of Chinese herbal medicine granules. This study used a metabolomics approach to validate the efficacy of herbal medicines, which can provide ideas for other studies.

## Mechanisms of Traditional Chinese Medicine in Treating Heart Failure

### Coronary Microvascular Dysfunction

Ischemic heart disease (IHD) commonly leads to HF (Pagliaro et al., 2020). Besides atherosclerosis and obstructive atherothrombotic events in epicardial coronary arteries, myocardial ischemia also has a close connection with coronary microvascular dysfunction (CMD) (Marzilli et al., 2012; Ford et al., 2018). CMD, characterized by coronary flow reserve (CFR) injury, means that the structure and function of coronary microcirculation are affected (Padro et al., 2020). As for obstructive coronary syndrome, although revascularization could recover the blood flow, CMD in distal coronary vasculature might bring about limited or incomplete recovery of damaged cardiomyocytes. Furthermore, myocardial fibrosis and adverse ventricular remodeling might result in HfrEF (Elgendy et al., 2019). In contrast to HFrEF resulting from myocardial scar and following left ventricular dilatation, the pathophysiological mechanisms of HFpEF are more complicated (Lam et al., 2018). As a result of CMD, heterogeneity in blood flow and oxygen levels would increase. Long term of CFR injury would result in damage to cardiac muscle cells and interstitial fibrosis (Yang et al., 2020). Thus, a wealth of supporting evidence suggests that CMD is the potential mechanism HFpEF and is a therapeutic target (Shah et al., 2018; Taqueti et al., 2018; Ahmad et al., 2021). The endothelium plays an important part in CMD. Endothelial structure and function are distinct in different angiosomes, whereas all of them could secrete and regulate vascular tension and substance permeability, especially in heart muscle capillaries, which are connected directly with endothelial cells and adjacent cardiomyocytes (Brutsaert, 2003; Zuchi et al., 2020). In addition, CMD is also associated with smooth muscle cell dysfunction, microvascular spasm/sympathetic dysfunction and altered microvascular remodeling (Pries and Reglin, 2017).

#### *Astragulus embranaceus* (*Fisch*.) *Bge*

Many TCM composite preparations include *Astragulus embranaceus* (*Fisch.*) *Bge.*, which could affect endothelial function. Further research of the effective ingredient in *Astragulus embranaceus* (*Fisch.*) *Bge*. also proved its function (Tse et al., 2012; Hu et al., 2018). For example, Astragaloside IV (PubChem CID: 13943297) can not only improve vascular endothelial dysfunction induced by hyperglycemia *via* inhibiting the Toll-like receptor 4 (TLR4)/nuclear factor kappa-B(NF-κB) signaling pathway (Leng et al., 2018), but also inhibits vascular endothelial dysfunction via the phosphatidylinositol 3-kinase (PI3K)/protein kinase B (AKT)/endothelial nitric oxide synthase (eNOS) signaling pathway in rat aortic rings (Lin et al., 2018). Calycosin (PubChem CID: 5280448) can also promote angiogenesis, *via* a mechanism that involves vascular endothelial growth factor (VEGF)-vascular endothelial growth factor receptor 2 (VEGFR2) and mitogen-activated protein kinase (MAPK) signaling pathways (Tang et al., 2010; Li et al., 2011). Astragalus polysaccharide (PubChem CID: 2782115) can restrain apoptosis of human cardiac microvascular endothelial cells undergoing hypoxia and reoxygenation by activating the PI3K/AKT signaling pathway (Xie et al., 2016), (Table 2).

**TABLE 2:** The mechanisms of TCM in improving CMD.

TCM	Component	PubChem CID	Structure	Underlying mechanisms	References
*Astragulus embranaceus (Fisch.) Bge*, 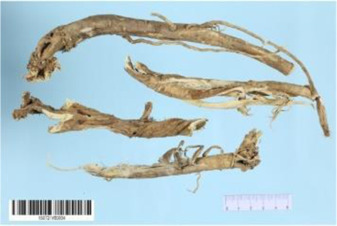	Astragaloside IV	13943297	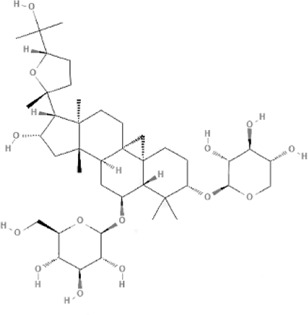	PI3K/AKT pathway↑	Leng et al. (2018)
				TLR4/NF-κB pathway↓	Lin et al. (2018)
	Calycosin	5280448	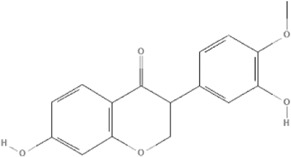	VEGF-VEGFR2 and MAPK pathways↑	Li et al. (2011)
					Tang et al. (2010)
	Astragalus polysaccharide	2782115	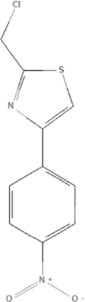	PI3K/AKT pathway↑	Xie et al. (2016)
*Panax ginseng C. A. Mey*, 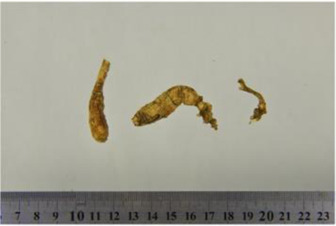	Ginsenoside Rg3	9918693	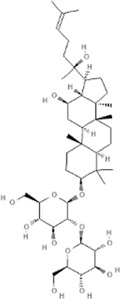	NF-κB↑	Kim et al. (2003)
	Ginsenoside Rg1	441923	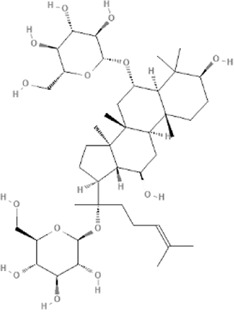	PI3K/AKT/p70S6K pathway ↑	Leung et al. (2011)
				NRF2-ARE pathway↑	Wang et al. (2015)
*Salvia miltiorrhiza Bge*, 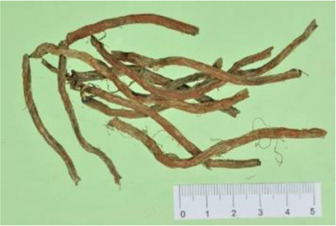	Tanshinone IIa	164676	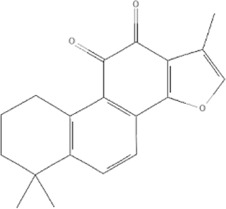	Estrogen receptor pathway↑	Cui et al. (2016)
				JAK2/STAT3 pathway↓	Fan et al. (2011)
				NRF2 pathway↑	He L et al. (2021)
	Ursolic acid	64945	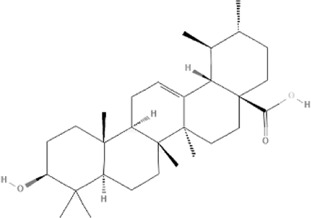	eNOS↑ and NOX4 ↓	Steinkamp-Fenske et al. (2007)
	Magnesium lithospermate B	6918234	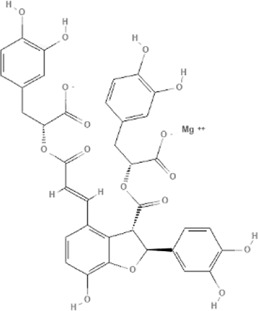	PI3K/AKT pathway↑	Liu Y. L et al. (2019)
*Panax notoginseng (Burk.) F. H. Chen*, 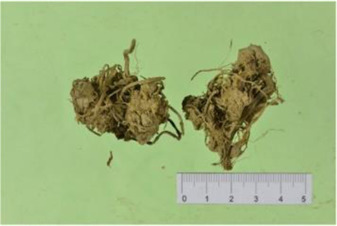	*Panax Notoginseng* saponins	NA	NA	SIRT1↓	Bo et al. (2020)
				AMPK Thr172 and CaMKII Thr287↑, eNOS dependent pathways↑	Wang et al. (2017a)
					Wang D et al. (2021)
	Notoginsenoside R1	441934	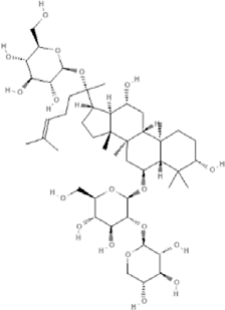	ANG2/TIE2 pathway↑	Fan et al. (2016)
				PI3K/AKT pathway↑	Fang et al. (2018)
				NRF2 pathway↑	Zhong et al. (2020)
*Ligusticum chuanxiong Hort*, 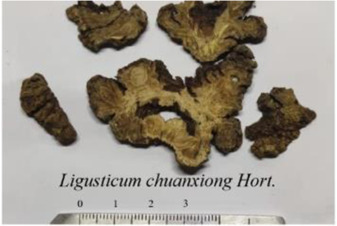	Tetramethylpyrazine	14296	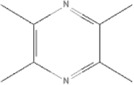	sEH↓, 14-3-3γ/BCL-2↑	Mak et al. (2017)
					Yang et al. (2019)
*Schisandra chinensis (Turcz.) Baill*, *Schisandra SphenantheraRehd.et Wils*, 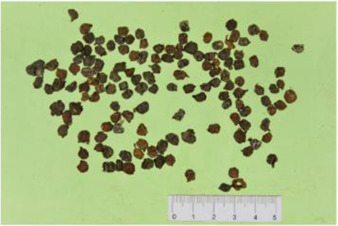	Gomisin A	634470	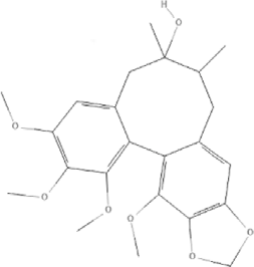	eNOS↑	Park et al. (2009)
*Bupleurum chinense DC*, 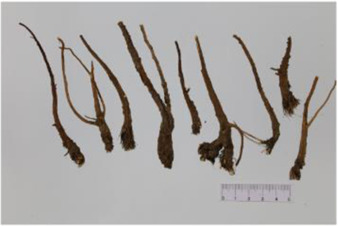	Saikosaponin C	131801344	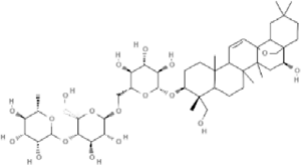	MMP-2↑, VEFG↑, MAPK↑, ERK↑	Shyu et al. (2004)

↑ (Upregulation), ↓ (Downregulation), 14-3-3γ (Tyrosine 3-monooxygenase/tryptophan 5-monooxygenase activation protein gamma), AKT (Protein kinase B), AMPK Thr172 (5′-adenosine monophosphat-activated protein kinase), ANG2 (Angiogenin 2), ARE (Antioxidant responsive element), BCL-2 (B-cell lymphoma-2), CaMKII Thr287 (Calcium/calmodulin-dependent protein kinase II), CMD (Coronary microvascular dysfunction), eNOS (Endothelial nitric oxide synthase), ERK (Extracellular-regulated kinase), JAK2 (Janus kinase 2), MAPK (Mitogen-activated protein kinase), MMP (Matrix metalloproteinase), NA (Not applicable), NF-κB (Nuclear factor kappa-B), NOX4 (NADPH oxidase 4), NRF (Nuclear factor-erythroid 2-related factor), p70S6K (Ribosomal protein S6 kinase B1), PI3K (Phosphatidylinositol 3-kinase), sEH (Soluble epoxide hydrolase ), SIRT1 (Sirtuin), STAT3 (Signal transducer and activator of transcription 3), TCM (Traditional Chinese Medicine), TIE2 (Tyrosine kinase with immunoglobin and epidermal growth factor homology domain 2), TLR4 (Toll-like receptor), VEGF (Vascular endothelial growth factor), VEGFR2 (Vascular endothelial growth factor receptor 2).

#### Panax ginseng C. A. Mey

Ginsenoside Rg3 (PubChem CID: 9918693) isolated from *Panax ginseng C. A. Mey.* undertakes key roles of relaxing vessels and exerting a cytoprotective effect via induction of inducible nitric oxide synthase (iNOS) by activating NF-κB (Kim et al., 2003). Rg3 can antagonize adriamycin-induced cardiotoxicity by improving endothelial dysfunction via upregulating the nuclear factor-erythroid 2-related factor-2 (NRF2)-antioxidant responsive element (ARE) pathway through activation of AKT (Wang et al., 2015). Under normal cellular oxygen conditions in human umbilical vein endothelial cells, Ginsenoside Rg1 (PubChem CID: 441923) is a valid stimulator of hypoxia-inducible factor, which is an important transcription regulator for numerous angiogenic factors, through activating PI3K/AKT/ribosomal protein S6 kinase B1 (p70S6K) signaling (Leung et al., 2011), (Table 2).

#### Salvia miltiorrhiza Bge

Studies on effective ingredients of *Salvia miltiorrhiza Bge.* are numerous. The direct vasorelaxation induced by Tanshinone IIa (PubChem CID: 164676) is mediated by the nongenomic action of the estrogen receptor through endothelial nitric oxide synthase activation and Ca^2+^ mobilization (Fan et al., 2011). Tanshinone IIa also attenuates hypoxia/reoxygenation (H/R)-induced apoptosis via inhibiting the Janus kinase 2 (JAK2)/signal transducer and activator of transcription 3 (STAT3) signaling pathway (Cui et al., 2016) and protects human coronary artery endothelial cells from ferroptosis by activating the NRF2 pathway (He L et al., 2021). Ursolic acid (PubChem CID: 64945) exerts beneficial effects by upregulation of eNOS and a parallel downregulation of nicotinamide adenine dinucleotide phosphate (NADPH) oxidase 4 (NOX4), leading to an increase in bioactive nitric oxide (NO) (Steinkamp-Fenske et al., 2007). Magnesium lithospermate B (PubChem CID: 6918234) also can activate eNOS, which in turn enhances vascular nitric oxide production, which is medicated *via* the PI3K/AKT pathway (Liu Y. L et al., 2019), (Table 2).

#### *Panax notoginseng* (*Burk*.) *F. H. Chen*

*Panax* notoginseng saponins, which are the major active components of *Panax notoginseng* (*Burk*.) *F. H. Chen*, are a kind of chemical mixture containing different dammarane-type saponins, such as Notoginsenoside R1, and Ginsenosides Rb1, Rd, Re, and Rg1 (Xu et al., 2019). The pro-angiogenic and endothelial protection effects of *Panax* notoginseng saponins have been demonstrated *in vitro* and *in vivo* experimental models by upregulating sirtuin 1 (SIRT1) and antioxidants, and enhancing autophagy through phosphorylation of 5′-adenosine monophosphate-activated protein kinase (AMPK) Thr172 and calcium/calmodulin-dependent protein kinase II (CaMKII) Thr287, and eNOS-dependent pathways (Wang D et al., 2017; Bo et al., 2020; Wang D et al., 2021). Notoginsenoside R1 (PubChem CID: 441934) plays an important role among Panax notoginseng saponin active component by activating the angiogenin 2 (ANG2)/tyrosine kinase with immunoglobin and epidermal growth factor homology domain 2 (TIE2) pathway (Zhong et al., 2020) and PI3K/AKT pathway (Fang et al., 2018) to promote angiogenesis and activating NRF2 in endothelial cells to suppressing monocyte adhesion (Fan et al., 2016), (Table 2).

#### Others

Tetramethylpyrazine (PubChem CID: 14296), the predominant active ingredient in *Ligusticum chuanxiong Hort.* or *Schisandra sphenantheraRehd.et Wils.*, can suppress angiotensin II-induced soluble epoxide hydrolase (sEH) expression in the coronary endothelium via an anti-endoplasmic reticulum (ER) stress mechanism (Mak et al., 2017) and attenuates the endotheliotoxicity of doxorubicin *via* tyrosine 3-monooxygenase/tryptophan 5-monooxygenase activation protein gamma (14-3-3γ)/B-cell lymphoma-2 (BCL-2) (Yang et al., 2019). Gomisin A (PubChem CID: 634470) contained in *Schisandra chinensis* (*Turcz*.) *Baill.* induces Ca^2+^-dependent activation of eNOS in human coronary artery endothelial cells, events that are linked to NO production and thereby endothelial-dependent vasorelaxation (Park et al., 2009). Saikosaponin C (PubChem CID: 131801344) in *Bupleurum chinense DC* can induce endothelial cell growth, migration, and capillary tube formation via activating MMP-2, VEGF, MAPK, and ERK (Shyu et al., 2004), (Table 2).

### Energy Metabolism

The heart is an organ with high energy demands. The adenosine-triphosphate (ATP) required for cardiac contraction, relaxation, and basal metabolism in healthy adults is mainly provided by mitochondrial oxidative phosphorylation, and a small part comes from glycolysis (Stanley et al., 2005). There are a variety of energy substrates that can be used by cardiomyocytes. Approximately 70–90% of cardiac ATP is a result of the oxidation of fatty acids and the remaining 10–30% comes from the metabolism of glucose, lactate, ketone bodies, and certain amino acids (Doenst et al., 2013). Changes in energy metabolism during HF are mainly related to changes in the metabolic substrates and mitochondrial changes (He Y et al., 2021). The metabolic changes of cardiomyocytes not only depend on the severity and type of HF, but also are related to different underlying diseases (Lopaschuk et al., 2021). Generally, in the early stage of HF, the utilization of fatty acids remains the same or increases slightly as the severity of HF progresses, the rate of myocardial fatty acid oxidation decreases (Bertero and Maack, 2018b). Glucose metabolism in HF is characterized by an increased glucose uptake and glycolysis rate, without accompanying increase in glucose oxidation (De Jong and Lopaschuk, 2017). In the late stage of HF, the reduced sensitivity of the myocardium to insulin can lead to impaired myocardial glucose uptake (Lin et al., 2017). Ketone bodies, as substrates of cardiometabolic metabolism, have received extensive attention in recent years (Selvaraj et al., 2020). The utilization of ketone bodies increases as the use of fatty acid and glucose decreases (Bedi et al., 2016). Clinical studies have shown that exogenous ketone body supplementation can improve heart function in patients with HF (Nielsen et al., 2019). Most of the ATP (about 95%) consumed by the heart comes from oxidative metabolism in the mitochondria (Manolis et al., 2021). Changes in metabolic substrates during HF mean that the function and structure of mitochondria also change (Bayeva et al., 2013; Chaanine et al., 2019). On the one hand, after mitochondrial damage, oxidative phosphorylation is reduced, and the production of high-energy phosphoric acid compounds in the respiratory chain is impeded, resulting in insufficient bioenergy and aggravating the progression of HF (Zhou and Tian, 2018). On the other hand, other changes in mitochondrial function and structure, such as impaired mitochondrial electron transport chain activity, increased formation of reactive oxygen species, aberrant mitochondrial dynamics, and altered ion homeostasis, are also closely related to the occurrence of HF (Brown et al., 2017).

#### Panax ginseng C. A. Mey

In the Langendorff system, total ginsenosides of *Panax ginseng C. A. Mey*. may modulate tricarboxylic acid cycle protein expression, such as L-lactate dehydrogenase B chain (LDHB), glycerol-3-phosphate dehydrogenase (G3PD), pyruvate dehydrogenase complex (PDC)and aldose reductase (AR), to improve cardiac energy metabolism in ischemic rat heart tissues (Wang et al., 2012). *Panax* ginseng polysaccharide protected mitochondria by inhibiting mitochondrial injury and swelling in a concentration-dependent manner, and improved energy metabolism (Li et al., 2009). The SIRT1 signaling pathway could improve glucose aerobic metabolism and mitochondrial biosynthesis and ginsenoside Rc acts as a SIRT1 activator (Huang et al., 2021). Ginsenoside Rg1 adjusts energy metabolism *via* regulating the expression of associated proteins, thus increasing the activity of mitochondria respiratory complexes and the expression of ATP synthase, H+ transporting, mitochondrial F1 complex, delta subunit (ATP5D), which might be the result of binding to Ras homolog family member A (RHOA) and inactivating RHOA/Rho associated coiled-coil containing protein kinase 1 (ROCK1) signaling (Li et al., 2018). Ginsenoside Rg1 an also promote autophagy, and inhibits apoptosis by weakening the interaction between Beclin1 and BCL-2 (Li et al., 2016). Ginsenoside Rb3(PubChem CID: 12912363) regulates energy metabolism and inhibits myocardial fibrosis by regulating peroxisome proliferator activated receptor *a* (PPARα) (Chen et al., 2019, Zhang Y et al., 2021). Ginsenoside Rb1(PubChem CID: 9898279) might regulate calcium signaling by alleviating protein O-GlcNAcylation to improve diabetic cardiomyopathy (Qin et al., 2019), (Table 3).

**TABLE 3:** The mechanisms of TCM in improving energy metabolism.

TCM	Component	PubChem CID	Structure	Underlying mechanisms	References
*Panax ginseng C. A. Mey* 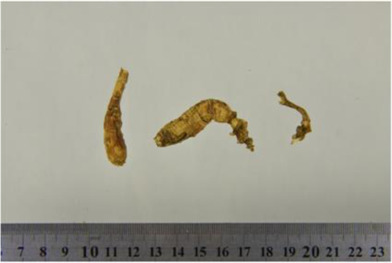	Total ginsenosides	NA	NA	LDHB↓, G3PD↓, PDC↓, AR↓, ATP↓	Wang et al. (2012)
	*Panax ginseng* polysaccharide	NA	NA	ATP↑, ADP↑, TAP↑, AEC↑	Li et al. (2009)
	Ginsenoside Rc	12855889	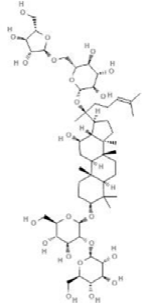	SIRT1 ↑	Huang et al. (2021)
	Ginsenoside Rg1	441923	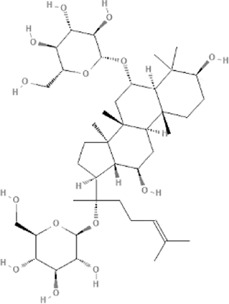	RHOA pathway↓, interaction between Beclin1 and BCL-2↓	Li et al. (2016)
					Li et al. (2018)
	Ginsenoside Rb3	12912363	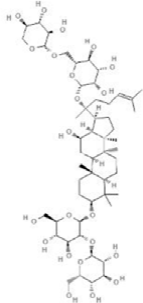	PPARα pathway↑	Chen et al. (2019)
					Zhang Y et al. (2021)
	Ginsenoside Rb1	9898279	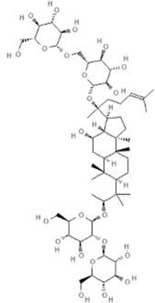	O-GlcNAcylation↓	Qin et al. (2019)
*Aconitum carmichaelii Debx*, 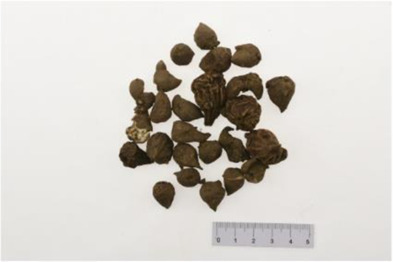	*Aconitum* alkaloids	NA	NA	Glucose↓, and increase AMP↑, GMP↑, ADP↑, ATP↑	Wu H et al. (2019)
	Salsolinol	91588	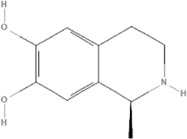	MCU pathway↓	Wen et al. (2019)
	Aconitine	245005	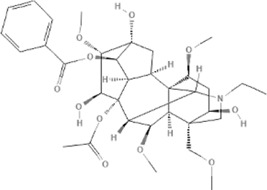	SIRT3↑ ,CYPD↓	Wang N. N et al. (2021)
*Astragulus embranaceus (Fisch.) Bge*, 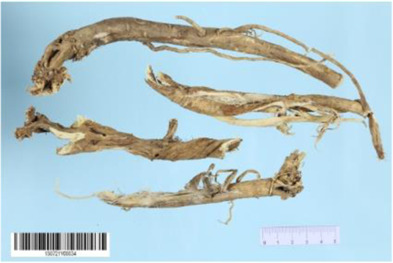	Astragaloside IV	13943297	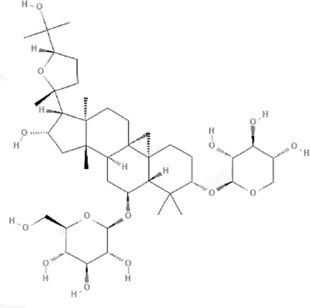	NF-κB/PGC1α pathway ↑, PPARα↑, MCAD↑, MCPT1↑, GSK-3beta↓, HES1↓, BCL-2-mediated mitochondrial function↑	Dong et al. (2017)
					He et al. (2012)
					Huang et al. (2016)
					Luo et al. (2019)
					Tang et al. (2018)
					Tu et al. (2013)
					Zhang et al. (2015)
	Formononetin	5280378	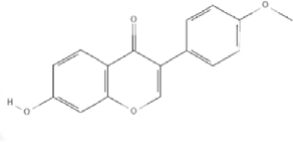	PPARγ pathway ↑	Nie et al. (2018)
	Astragalus polysaccharide	2782115	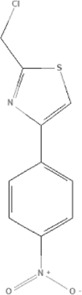	TNF-α/PGC1α pathway ↑	Luan et al. (2015)
*Salvia miltiorrhiza Bge*, 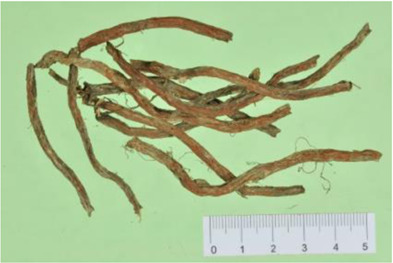	Tanshinone IIa	164676	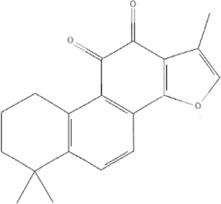	SIRT1/PGC1α pathway↑	Zhong et al. (2019)
	Cryptotanshinone	160254	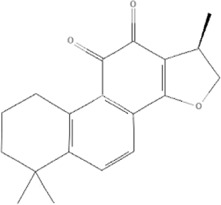	PGC1α↑, NRF-1↑, TFAM↑	Zhang et al. (2016)

↑(Upregulation), ↓(Downregulation), ADP (Adenosine diphosphate), AEC (Adenylate energy charge), AR (Aldose reductase), AMP (Adenosine monophosphate), ATP (Adenosine-triphosphate), BCL-2 (B-cell lymphoma-2), CYPD (Cyclophilin D), G3PD (Glycerol-3-phosphate dehydrogenase), GMP (Guanosine monophosphate), GSK-3beta (Glycogen synthase kinase 3 beta), HES1 (Hairy and enhancer of split-1), LDHB (L-lactate dehydrogenase B chain), MCAD (Medium-chain acyl-CoA dehydrogenase), MCPT1 (Mast cell protease 1), MCU (Mitochondrial calcium uniporter), NA (Not applicable), NF-κB (Nuclear factor kappa-B), NRF-1 (Nuclear factor-erythroid 2-related factor), PDC (Pyruvate dehydrogenase complex), O-GlcNAcylation (O-linked β--acetylglucosamine modification), PGC1α (Peroxisome proliferator-activated receptor gamma coactivator-1 alpha), PPAR (Peroxisome proliferator-activated receptor), RHOA (Ras homolog family member A), SIR (Sirtuin), TAP (Total adenylate pool), TCM (Traditional Chinese Medicine), TFAM (Transcription factor A, mitochondrial), TNF-α (Tumor necrosis factor-α).

#### Aconitum carmichaelii Debx

*Aconitum carmichaelii Debx* is a TCM that is, used extensively in HF as the processed product of *Aconitum carmichaelii Debeaux* tubers (Zhou W et al., 2021). *Aconitum* alkaloids, which include C19-diterpenoid alkaloids (mainly comprising aconitine, mesaconitine, and hypaconitine) and C20-diterpenoid alkaloids (predominantly benzoylmesaconitine, benzoylaconitine, and benzoylhypacoitine) and other alkaloids (mainly comprising higenamine and salsolinol) have versatile chemical structures (Zhou et al., 2015). Their cardiotoxicity and neurotoxicity hinder the use of Aconitum alkaloids; however, in TCM’s theory that even toxic substances are powerful medicines and can show reduced toxicity by proper methods (Liu et al., 2017; Zong et al., 2019; Mi et al., 2021). Emerging evidence also shows that *Aconitum* alkaloids could improve energy metabolism and mitochondrial function to generate cardioprotective effects. *Aconitum* alkaloids could reduce glucose levels, and increase creatine, adenosine monophosphate (AMP), Guanosine monophosphate (GMP), Adenosine diphosphate (ADP), and ATP levels in myocardial infarction rats (Wu H et al., 2019). Salsolinol (PubChem CID: 91588) attenuates doxorubicin-induced CHF in rats via a mechanism that might be related to the mitochondrial calcium uniporter (MCU) pathway (Wen et al., 2019). Aconitine (PubChem CID: 245005) alleviated the energy metabolic dysfunction of a myocardial injury model induced by Angiotensin II in H9c2 cells by activating SIRT3 to deacetylate cyclophilin D (CYPD) (Wang N. N et al., 2021). A combination of *Zingiber officinale Rosc.* and A*conitum carmichaelii Debx*. showed an enhancing effect (Yu et al., 2012; Wen et al., 2020a). The potential mechanism of this effect might be related to mitochondrial energy metabolism, and it can be mediated by MCU in AHF rats induced by propafenone hydrochloride (Zhang et al., 2017) and improved by the liver kinase B1 (LKB1)/AMPKα/SIRT1 signaling pathway in doxorubicin (DOX)-induced CHF rats (Wen et al., 2020b), (Table 3).

#### *Astragulus embranaceus* (*Fisch*.) *Bge*

Astragaloside IV can increase the ratio of ATP/AMP, and decrease the content of FFAs in heart tissue of rats compared with isoproterenol alone *via* NF-κB/peroxisome proliferator-activated receptor gamma coactivator-1 alpha (PGC1α) signaling-mediated energy biosynthesis (Zhang et al., 2015). The function of Astragaloside IV in improving fatty acid utilization might be connected with PPARα, medium-chain acyl-CoA dehydrogenase (MCAD), and mast cell protease 1 (MCPT1) (Dong et al., 2017; Tang et al., 2018). Astragaloside IV prevents ischemia/reperfusion (I/R) injury by maintaining the mitochondrial membrane potential, inhibiting mitochondrial permeability transition pore opening, and improving energy metabolism via inactivating glycogen synthase kinase 3 beta (GSK-3beta) and hairy and enhancer of split-1 (HES1), thus improving BCL-2-mediated mitochondrial function (He et al., 2012; Tu et al., 2013; Huang et al., 2016; Luo et al., 2019). Formononetin (PubChem CID: 5280378) has beneficial effects on obesity and insulin resistance by modulating PPARγ activity (Nie et al., 2018). Astragalus polysaccharide attenuates iso-induced cardiac hypertrophy through regulating tumor necrosis factor-α (TNF-α)/PGC1α signaling, resulting in decreased FFA contents (Luan et al., 2015), (Table 3).

#### Salvia miltiorrhiza Bge

Tanshinone IIa blocks mitochondrial damage *via* activating the SIRT1/PGC1α pathway in acute cardiac microcirculation I/R injury (Zhong et al., 2019). Cryptotanshinone (PubChem CID: 160254) protects against adriamycin-induced mitochondrial dysfunction in cardiomyocytes by increasing the activities of complexes, except complex II, and promotes mitochondrial biogenesis-relative factors PGC1α, NRF-1, and TFAM (Zhang et al., 2016), (Table 3).

### Oxidative Stress

Oxidative stress is defined as an imbalance between the generation of reactive oxygen species (ROS) and the endogenous antioxidant defense mechanisms (Zhou Y et al., 2021). At low concentrations, ROS modulates critical functions in cell homeostasis, whereas an overabundance of ROS plays a crucial role of worsening of the systolic and diastolic function and HF (Munzel et al., 2015). ROS production in the heart is primarily achieved by the mitochondria, NADPH oxidases, xanthine oxidase, and uncoupled nitric oxide synthase (NOS) (Munzel et al., 2017). Although pre-clinical studies have shown the beneficial effects of antioxidant strategies on HF, clinical studies in patients with HF have not achieved ideal results. A meta-analysis of 50 RCTs showed that supplementation with vitamins and antioxidants was not associated with reductions in the risk of major cardiovascular events (Myung et al., 2013). The future of anti-oxidative stress therapies might lie in increasing the endogenous antioxidant capacity or targeting mitochondrial ROS (Bertero and Maack, 2018a; Zhang et al., 2020). Study of TCM in oxidative stress might provide new ideas for the antioxidant treatment of HF (Table 3).

#### Coptis chinensis Franch

Berberine (PubChem CID: 2353), Palmatine (PubChem CID: 19009), and Coptisine (PubChem CID: 72322) are the main components of *Coptis chinensis Franch.* (Meng et al., 2018). Berberine protects rat hearts from I/R injury by decreasing the level of ROS and malondialdehyde (MDA), improving mitochondrial dysfunction via increasing MMP and complex I activity, as well as activating the SIRT1/tumor protein p53 (p53) signaling pathway (Liu D. Q et al., 2019). Berberine also ameliorates the doxorubicin-induced oxidative insult and mitochondrial damage as a modulator of SIRT3 or the SIRT1/Src-homology-2-domain-containing transforming protein 1 (p66Shc)-mediated pathway (Coelho et al., 2017; Wu Y. Z et al., 2019). Coptisine reduces oxidative stress by activating AKT and the JNK/NRF2/NAD(P)H quinone dehydrogenase 1 (NQO1) pathway (Hu et al., 2017), and Palmatine can inhibit the increase of lactate dehydrogenase (LDH), Creatine kinase (CK), and MDA and halt the decline of superoxide dismutase (SOD) and catalase (CAT) in I/R cardiac tissues (Kim et al., 2009), (Table 4).

**TABLE 4:** The mechanisms of TCM in improving oxidative stress.

TCM	Component	PubChem CID	Structure	Underlying mechanisms	References
*Coptis chinensis Franch*, 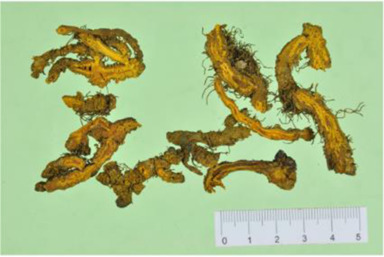	Berberine	2353	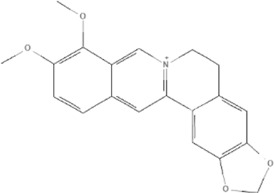	SIRT1/p53 pathway, SIRT3↑, SIRT1/p66Shc pathway↑	Coelho et al. (2017)
					Liu D. Q et al. (2019)
					Wu Y. Z et al. (2019)
	Coptisine	72322	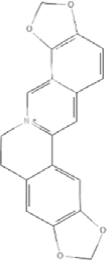	AKT and JNK/NRF2/NQO1 pathway↑	Hu et al. (2017)
	Palmatine	19009	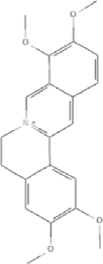	LDH↓, CK↓, MDA↓, SOD↓, and CAT↓	Kim et al. (2009)
*Rhodiola crenulate (Hook .f. et Thoms.) H. Ohba*, 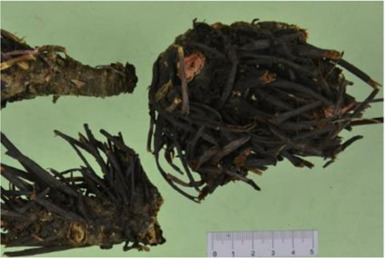	Salidroside	159278	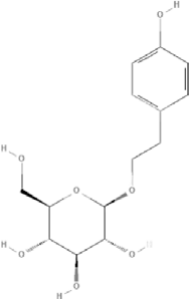	PI3K-AKT pathway↑, NOX1↓, mTOR↑, AMPK↑, AKT/HO-1↑	Hao et al. (2021)
					Ni et al. (2021)
					Wang et al. (2013)
					Zhu et al. (2011)
*Polygonum cuspidatum Sieb. et Zucc*, 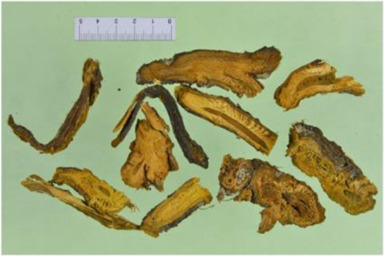	Polydatin	445154	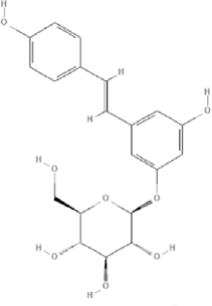	NADPH↓, NF-κB↓	Dong et al. (2015)
				Notch1/HS1- PTEN/AKT↑,ROCK↓	Tan et al. (2020)
					Yu et al. (2018)
	Resveratrol	5281718	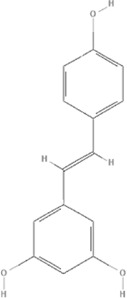	DJ-1↑, mitochondrial complex I ↑, HMGB1 pathway↓, SIRT1↑, AMPK pathway↑, autophagy by SIRT1/FOXO1/RAB7 axis↑	Bagul et al. (2015)
					Guo et al. (2015)
					Wang et al. (2014)
					Wu et al. (2016)
					Zhang et al. (2018)
*Curcuma Longa L* 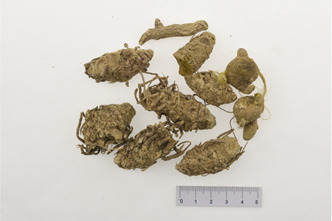	Curcumin	969516	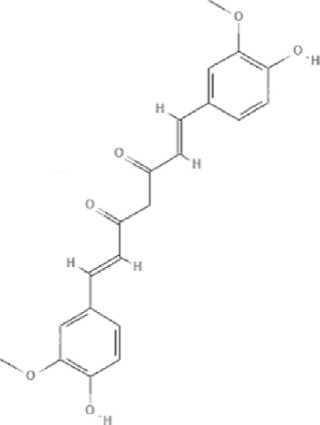	mTOR or 14-3-3γ pathway↑, SIRT1↑, NRF2↑, NF-κB↓, SIRT1-FOXO1 and PI3K-AKT↑	He et al. (2018)
					Liu Q et al. (2019)
					Ren et al. (2020)
					Xiao et al. (2016)
					Yu et al. (2020)
					Zeng et al. (2015)

↑ (Upregulation), ↓ (Downregulation), 14-3-3γ (Tyrosine 3-monooxygenase/tryptophan 5-monooxygenase activation protein gamma) AKT (Protein kinase B) AMPK (5′-adenosine monophosphat-activated protein kinase), CAT(Catalase), CK (Creatine kinase), DJ-1 (Parkinsonism associated deglycase), FOXO1 (Forkhead box O1), HMGB1 (High-mobility group box 1), HO-1 (Heme oxygenase-1), HS1 (Heat stable protein 1), JNK (c-Jun N-terminal kinase), LDH (Lactate dehydrogenase), MDA (Malondialdehyde), mTOR (Mechanistic target of rapamycin), NADPH (Nicotinamide Adenine Dinucleotide Phosphate), NF-κB (Nuclear factor kappa-B), Notch1 (Notch receptor 1), NOX (NADPH oxidase), NQO1 [NAD(P)H quinone dehydrogenase 1], NRF2 (Nuclear factor-erythroid 2-related factor), p53 (Tumor protein p53), p66Shc (Src-homology-2-domain-containing transforming protein 1), PI3K (Phosphatidylinositol 3-kinase), PTEN (Phosphatase and tensin homolog), RAB7 (Member RAS oncogene family), ROCK (Rho associated coiled-coil containing protein kinase 1), SIRT(Sirtuin), SOD (Superoxide dismutase), TCM (Traditional Chinese Medicine).

#### *Rhodiola crenulate* (*Hook. f. et Thoms*.) *H. Ohba*

Salidroside (PubChem CID: 159278) has been identified as one of the most active ingredients isolated from *Rhodiola crenulate (Hook.f. et Thoms.) H. Ohba*, which reduces oxidative stress to fight against cardiovascular diseases (Tao et al., 2019; Zhao et al., 2021). Salidroside protected cardiomyocytes against hydrogen peroxide-induced injury *via* an PI3K-AKT dependent pathway and increaed the expression and activities of endogenous PI3K-dependent antioxidant enzymes (Zhu et al., 2011). In doxorubicin-induced cardiotoxicity, salidroside suppressed the excessive oxidative stress by inhibiting NOX1 and augmented the endogenous antioxidative enzymes, catalase, and manganese superoxide dismutase (Wang et al., 2013). Salidroside protects against diabetes-induced cardiac dysfunction by modulating the mechanistic target of rapamycin (mTOR), AMPK, and AKT/heme oxygenase-1(HO-1) signaling pathways (Hao et al., 2021; Ni et al., 2021), (Table 4).

#### Polygonum cuspidatum Sieb. et Zucc

Over 67 compounds been isolated from *Polygonum cuspidatum Sieb. et Zucc*. and identified, among them, Polydatin (PubChem CID: 5281718) and Resveratrol (PubChem CID: 445154) have attracted wide attention in the field of oxidative stress (Peng et al., 2013). Polydatin protects myocardial function in diabetic rats *via* inhibition of NADPH oxidase and NF-κB activities (Tan et al., 2020), and also alleviates oxidative/nitrative stress damage *via* the Notch receptor 1 (Notch1)/heat stable protein 1(HS1)-phosphatase and tensin homolog (PTEN)/AKT signaling pathway in diabetic myocardial I/R injury (Yu et al., 2018). Polydatin prevents myocardial hypertrophy mediated by inhibition of ROS-dependent ROCK activation (Dong et al., 2015). In models of cardiac I/R injury, pharmacological agent-induced cardiotoxicity, obesity, long-term nicotine intake, and diabetes, Resveratrol activates the antioxidant genes such as those encoding HO-1, SOD, CAT, and glutathione (GSH), which can help to promote the balance between oxidative stress and antioxidants, especially in the mitochondria (Kovacic and Somanathan, 2010; Li et al., 2012; Ahmad and Hoda, 2020; Ramalingam et al., 2021). Parkinsonism associated deglycase (DJ-1) preserves mitochondrial complex I activity, thus playing an important role in Resveratrol-mediated cardioprotective effects against I/R-induced oxidative stress damage (Zhang et al., 2018). For the myocardial damage caused by diabetes, Resveratrol can inhibit the high-mobility group box 1tbox1 (HMGB 1)-mediated signaling pathway (Wu et al., 2016), activate SIRT1 leading to deacetylation of both NF-kB/p65 and histone 3 (H3) (Bagul et al., 2015), improve AMPK-related pathways (Guo et al., 2015), and enhance autophagy *via* the SIRT1/Forkhead box O1(FOXO1)/member RAS oncogene family (RAB7) axis (Wang et al., 2014), (Table 4).

#### Curcuma Longa L

Curcumin (PubChem CID: 969516) is an important compound in *Curcuma Longa L*., which is the golden spice in treating cardiovascular diseases (Li et al., 2020). Chemotherapeutic drugs induce cardiotoxicity, limiting their clinical application, and curcumin rescues DOX-induced cardiac injury by suppressing oxidative stress and improving mitochondrial function via regulation of the mode of cell death (autophagy, apoptosis, and pyroptosis) *via* an mTOR or 14-3-3γ-dependent pathway (He et al., 2018; Liu Q et al., 2019; Yu et al., 2020). Curcumin acts as an agonist of SIRT1 to protect against myocardial infarction-induced cardiac fibrosis (Xiao et al., 2016). The protection provided by curcumin in myocardial damage induced by metabolic disorders might be associated with activating Nrf2, inactivating NF-κB, and modulating the SIRT1-FOXO1 and PI3K-AKT pathways (Zeng et al., 2015; Ren et al., 2020), (Table 4).

### Inflammation

The innate immune system is activated by a variety of cardiac disease states that lead to cardiac injury through the interaction between damage-associated molecular patterns (DAMPs) or pathogen-associated molecular patterns (PAMPs) and pattern-recognition receptors (PRRs), most commonly TLR4 (Dutka et al., 2020). Activation of PRRs induces a variety of non-cellular effectors (pro-inflammatory cytokine, chemokines, and inflammasome assembly) and cellular effectors (neutrophils, monocytes, and macrophages) in the heart, especially NF-kB (Adamo et al., 2020). Adaptive immunity is activated by the innate immune system, resulting in the recruitment of B cells and T cells to injured cardiomyocytes (Rhee and Lavine, 2020). On a myocardial level, inflammation promotes myofibroblast formation and interstitial collagen deposition, and influences multiple peripheral organ systems to exacerbate the development of HF (Murphy et al., 2020). Similar to the clinical studies of oxidative stress, the results of other anti-inflammatory treatments are not satisfactory, except in the CANTOS trial (Everett et al., 2020). The study of inflammation in TCM might provide new ideas for the treatment of HF.

#### Genus *Paeonia*

*Paeonia lactiflora Pall.* and *Paeonia veitchii Lynch* belong to the genus *Paeonia*, from which more than 400 compounds have been structurally identified (Yan et al., 2018; Li P et al., 2021). Paeoniflorin (PubChem CID: 442534) is unique to the genus *Paeonia* and several studies have reported its anti-inflammatory effects (Jiao et al., 2021). Paeoniflorin decreased the levels of tumor necrosis factor-α (TNFα) and interleukin-1β (IL)-1β in a mouse pressure overload-induced cardiac remodeling model by inhibiting NF-κB pathways (Zhou et al., 2013). Paeoniflorin reduces TNFα, IL-6, and monocyte chemoattractant protein (MCP)-1 levels and plays a cardioprotective role in spontaneous hypertensive rats *via* the MAPK signaling pathway (Liu X et al., 2019). The levels of inflammatory cytokines of TNF-α, IL-1β, IL-6, IL-12, MCP-1, and interferon (IFN)-γ can be decreased by paeoniflorin in endotoxemic mice to improve cardiac function via activation of PI3K/AKT signaling (Zhai and Guo, 2016). Gallic acid is a tannin of the genus *Paeonia*. Gallic acid (PubChem CID: 370) protects cardiac dysfunction by reducing the level of IL-6 and TNF-α in particulate matter-induced rat model (Radan et al., 2019) and ameliorates angiotensin II-induced atrial fibrillation by inhibiting immunoproteasome-mediated PTEN degradation in mice (Han D et al., 2020). Paeonol (PubChem CID: 11092) is another compound in the genus *Paeonia*, especially *Paeonia suffruticosa Andr.*, which has a cardioprotective effect in epirubicin-induced cardiotoxicity *via* increasing MicroRNA-1 (miR-1) to suppress the PI3K/AKT/mTOR and NF-kB signaling pathways (Wu et al., 2018) and reducing inflammatory damage in I/R injury rats (Ma et al., 2016), (Table 5).

**TABLE 5:** The mechanisms of TCM in improving inflammation.

TCM	Component	PubChem CID	Structure	Underlying mechanisms	References
*Paeonia lactiflora Pall*, 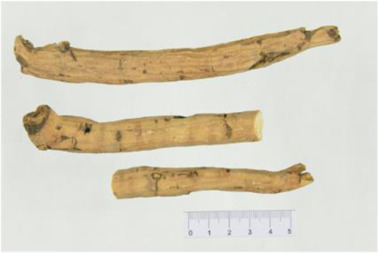	Paeoniflorin	442534	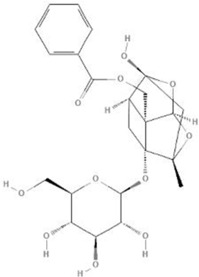	NF-κB pathway↓	Liu X et al. (2019)
				MAPK pathway↓	, Zhai and Guo, (2016),
				PI3K/AKT pathway ↑	Zhou et al. (2013)
*Paeonia veitchii Lynch*, 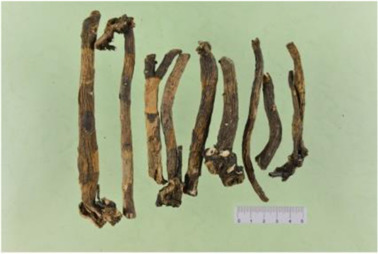	Gallic acid	370	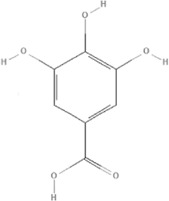	PTEN↑	Han D et al. (2020)
*Paeonia suffruticosa Andr*, 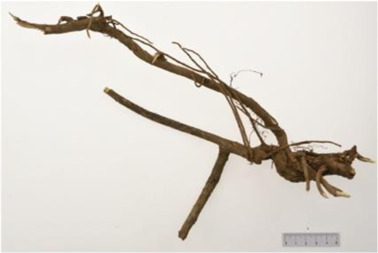	Paeonol	11092	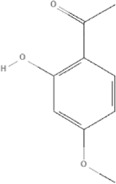	miR-1↑	Wu et al. (2018)
*Crataegus pinnatifida Bge. var. major N.E.Br*, 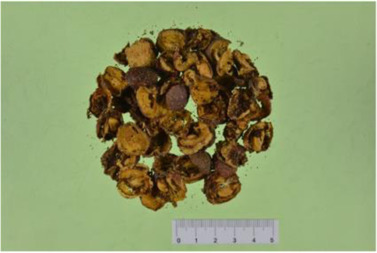	Hyperoside	5281643	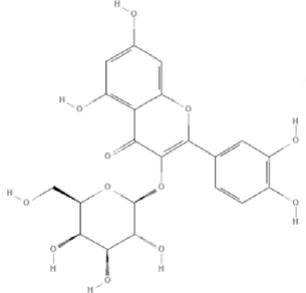	p38 MAPK, JNK, ERK and NF-κB pathway↓, NLRP1 inflammation pathway↓, AKT pathway ↓, miR-21↓	Jang et al. (2018)
					Ku et al. (2014)
					Wang X et al. (2018)
					Yang et al. (2021)
					Zhang J et al. (2021)
*Cinnamomum cassia Presl*, 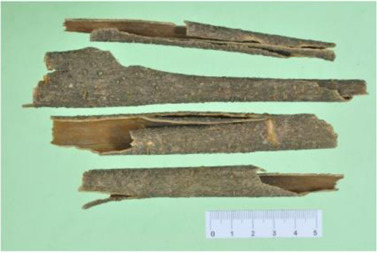	2-methoxycinnamaldehyde	641298	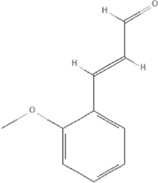	HO-1↑	Hwa et al. (2012)
	Cinnamaldehyde	637511	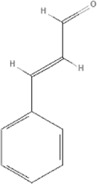	TLR4/6-IRAK4/1 signal↓, TLR4-NOX4 pathway↓, TLR4-NF-κB ↓	Ding et al. (2010)
					Kang et al. (2016)
					Song et al. (2013)
					Zhao et al. (2016)

↑ (Upregulation),↓ (Downregulation), AKT (Protein kinase B), ERK (Extracellular-regulated kinase), HO-1 (Heme oxygenase-1), IRAK (IL-1R-associated kinase), JNK (c-Jun N-terminal kinase), MAPK (Mitogen-activated protein kinase), miR (MicroRNA), NF-κB (Nuclear factor kappa-B),NLRP1 (Nucleotide-binding domain and leucine-rich repeat related family, pyrin domain containing 3), NOX (NADPH oxidase), p38 MAPK (Mitogen-activated protein kinase), PI3K (Phosphatidylinositol 3-kinase), PTEN (Phosphatase and tensin homolog ), TCM (Traditional Chinese Medicine), TLR (Toll-like receptor).

#### Crataegus pinnatifida Bge. var. Major N.E.Br

Hyperoside (PubChem CID: 5281643), a flavonoid from *Crataegus pinnatifida Bge. var. major N.E.Br.*, suppresses vascular inflammatory responses in diabetic complications and atherosclerosis by downregulating mitogen-activated protein kinases (p38 MAPK, JNK, and ERK) and NF-κB signaling (Ku et al., 2014; Jang et al., 2018). Hyperoside also plays a protective role against heart damage caused by other diseases. Hyperoside ameliorated myocardial hypertrophy, collagen volume fraction, and cardiomyocyte inflammation in the myocardial infarction mice by regulating autophagy via the nucleotide-binding domain and leucine-rich repeat related (NLR) family, pyrin domain containing 1(NLRP1) inflammation pathway (Yang et al., 2021). Hyperoside decreased the levels of inflammatory factors, including IL-1β, IL-6, TNF-α, and MCP-1 in an angiotensin II-induced cardiomyocyte hypertrophy model to improve cardiac function *via* AKT signaling (Wang X et al., 2018). Hyperoside treated cardiac dysfunction in a mouse model of sepsis by regulating cardiomyocyte viability and inflammation *via* suppressing miR-21 (Zhang J et al., 2021), (Table 5).

#### Cinnamomum cassia Presl

The anti-inflammatory effects of *Cinnamomum cassia Presl* extracts have been used to treat a variety of diseases, and research on its anti-inflammatory mechanism has found that it might be closely related to NF-κB, and Toll-like receptors TLR2 and TLR4 (Reddy et al., 2004; Schink et al., 2018). More than 160 components have been isolated and identified from *Cinnamomum cassia Presl*. Phenylpropanoids are the main bioactive components, including 2-Methoxycinnamaldehyde (PubChem CID: 641298), Cinnamaldehyde (PubChem CID: 637511) (Zhang et al., 2019). 2-Methoxycinnamaldehyde acid inhibits vascular cell adhesion molecule-1(VCAM-1) and NF-κB expression, which are activated by TNF-α in endothelial cells and inhibited by HO-1 induction, thus the heart function of rats with I/R injury can be protected (Hwa et al., 2012). Cinnamaldehyde inhibits the activation of NLRP3 inflammasomes by attenuating the cluster of differentiation 36 (CD36)-mediated TLR4/6-IRAK (IRAK) 4/1 signaling, thereby reducing fructose-induced cardiac inflammation and fibrosis (Kang et al., 2016). Cinnamaldehyde can also improve lipopolysaccharide-induced cardiac dysfunction through the TLR4-NOX4 pathway (Zhao et al., 2016). Cinnamaldehyde and its derivative cinnamic acid can reduce TNF-α and IL-6 in rats with acute myocardial ischemia induced by isoproterenol (Song et al., 2013), which also directly reduces the inflammation of viral myocarditis induced by coxsackie virus B3 by inhibiting the TLR4-NF-κB signal transduction pathway (Ding et al., 2010), (Table 5).

## Discussion

In this review, we retrospectively analyzed clinical studies on the intervention of TCM in HF, and discussed the mechanisms of some commonly used TCMs and their components in the treatment of HF. From the perspective of clinical research, TCM has the advantages of good curative effect and low levels of side effects in the treatment of HF, which can make up for the shortcomings of current treatment methods to a certain extent, and the two sides can produce complementary advantages. Basic research has shown that TCM can play its role in many ways, such as microcirculation improvements, energy metabolism promotion, anti-inflammation, and anti-oxidation. However, it should be noted that the compositions of TCM are complex. On the one hand, a variety of chemical components can affect organisms through many biological reactions. On the other hand, this kind of diversity makes it possible for different active ingredients in TCM to be synergistic, enhancing, and antagonistic. In the basic theories of TCM, the principles of drug application are also mentioned. However, currently, it is unclear which combinations of active ingredients have synergistic and antagonistic effects, or which combinations might increase toxicity, not to mention the effective or optimal dose of each active ingredient in the combination.

HF comprises a group of clinical syndromes with complex pathological mechanisms, involving multiple signaling pathways and targets. Natural medicines or their active ingredients can act on a variety of pathways and targets to effectively treat diseases, which is also the advantage of the application of TCM. To clarify the mechanism of TCM, it is necessary to study the pathways and targets of each active ingredient alone and in different combinations. It is also necessary to find a suitable breakthrough point and establish a reasonable pharmacological model based on genomics, proteomics, functional metabolomics, TCM pharmacology, and other -omics research (Han Y et al., 2020; Ma et al., 2020; Wang T et al., 2021). Determining the mechanism of the effective ingredients can not only explain how the various ingredients in TCM work individually or in combination, but also, and more importantly, it can discover new mechanisms and synergistic effects of the effective ingredients, which is conducive to innovation of TCM and the development of TCM theories.

A large number RCTs of integrated TCM and western medicine are reported every year; however, the quality of these studies is uneven. On the one hand, clinical research in TCM should carry out randomized, double-blind, placebo-controlled large-sample, multi-center RCT studies. During the implementation of RCTs, patients need to be included in strict accordance with the latest diagnostic criteria. The design of the placebo should be completely consistent with TCM in appearance and smell etc. Curative effect indicators should pay more attention on hard endpoints of cardiovascular events or choose internationally recognized intermediate endpoints as the primary endpoints to carry out long-term follow-up. On the other hand, the advantage of TCM treatment lies in individualized therapy based on syndrome differentiation. The direction of our future efforts is to form an RCT research method for TCM treatment and enable TCM with RCT evidence to enter clinical practice.

## Conclusion

The results of RCTs indicate that as an adjuvant treatment to conventional drugs, TCM might be beneficial to patients with HF. Recent studies on the mechanism of HF *in vitro* and in animal models have shown that TCM has microcirculation improvement, energy metabolism promotion, anti-inflammation, and anti-oxidation effects.

## Glossary

14-3-3γ: Tyrosine 3-monooxygenase/tryptophan 5-monooxygenase activation protein gamma
6MWD: 6-min walking distance
ADP: Adenosine diphosphate
ADRs: Adverse drug reactions
AEC: Adenylate energy charge
AEs: Adverse events
AHF: Acute heart failure
AKT: Protein kinase B
AMP: Adenosine monophosphate
AMPK: 5′-adenosine monophosphat-activated protein kinase
ANG2: Angiogenin 2
AR: Aldose reductase
ARE: Antioxidant responsive element
ATP: Adenosine-triphosphate
ATP5D: ATP synthase, H+ transporting, mitochondrial F1 complex, delta subunit
BCAAs: Branched-chain amino acids
BCL-2: B-cell lymphoma-2
BNP: Brain natriuretic peptide
CaMKII: Calcium/calmodulin-dependent protein kinase II
CAT: Catalase
CCEs: Composite cardiac events
CD36: Cluster of differentiation 36
CFR: Coronary flow reserve
CHF: Chronic heart failure
CHFQLS: Quality of Life measured by the CHF Integrated Chinese and Western Medicine Survival Scale
CK: Creatine kinase
CM: Chinese medicine
CMD: Coronary microvascular dysfunction
CYPD: Cyclophilin D
DAMPs: Damage-associated molecular patterns
DJ-1: Parkinsonism associated deglycase
DOX: Doxorubicin
ECG: Electrocardiograph
eNOS: Endothelial nitric oxide synthase
ERK: Extracellular-regulated kinase
FFAs: Free fatty acids
FOXO1: Forkhead box O1
G3PD: Glycerol-3-phosphate dehydrogenase
GMP: Guanosine monophosphate
GSH: Glutathione
GSK-3beta: Glycogen synthase kinase 3 beta
H/R: Hypoxia/reoxygenation
H3: Histone 3
HES1: Hairy and enhancer of split-1
HF: Heart Failure
HFmrEF: HF with mildly reduced ejection fraction
HFpEF: HF with preserved ejection fraction
HFrEF: HF with reduced ejection fraction
HMGB1: High-mobility group box 1
HO-1: Heme oxygenase-1
HS1: Heat stable protein 1
I/R: Ischemia/reperfusion
IFN: Interferon
IHD: Ischemic heart disease
IHF: Ischemic heart failure
IL: Interleukin
iNOS: Inducible nitric oxide synthase
INTER-CHF: INTERnational Congestive Heart Failure
IRAK: IL-1R-associated kinase
JAK2: Janus kinase 2
JNK: c-Jun N-terminal kinase
LA: Lactic acid
LDH: Lactate dehydrogenase
LDHB: L-lactate dehydrogenase B chain
LKB1: Liver kinase B1
LPE: Lysophosphatidylethanolamine
LVED: Left ventricular end-diastolic diameter
LVEDD: Left ventricular end diastolic diameter
LVEF: Left ventricular ejection fraction
LVIDd: Left ventricular internal diastolic diameter
LVIDs: Left ventricular internal dimension systole
MAPK: Mitogen-activated protein kinase
MCAD: Medium-chain acyl-CoA dehydrogenase
MCP: Monocyte chemoattractant protein
MCPT1: Mast cell protease 1
MCU: Mitochondrial calcium uniporter
MDA: Malondialdehyde
MI: Myocardial infarction
miR: MicroRNA
MLHFQ: Minnesota Living with Heart Failure Questionnaire
MMP: Matrix metalloproteinase
mTOR: Mechanistic target of rapamycin
NADPH: Nicotinamide Adenine Dinucleotide Phosphate
NF-κB: Nuclear factor kappa-B
NLRP3: Nucleotide-binding domain and leucine-rich repeat related family, pyrin domain containing 3
NO: Nitric oxide
NOS: Nitric oxide synthase
Notch1: Notch receptor 1
NOX: NADPH oxidase
NQO1: NAD(P)H quinone dehydrogenase 1
NRF: Nuclear factor-erythroid 2-related factor
NT-proBNP: N-terminal pro-B-type natriuretic peptide
NYHA: New York Heart Association
O-GlcNAcylation: O-linked β--acetylglucosamine modification
p53: Tumor protein p53
p66Shc: Src-homology-2-domain-containing transforming protein 1
p70S6K: Ribosomal protein S6 kinase B1
PA: Pyroracemic acid
PAMPs: Pathogen-associated molecular patterns
PDC: Pyruvate dehydrogenase complex
PGC1α: Peroxisome proliferator-activated receptor gamma coactivator-1 alpha
PI3K: Phosphatidylinositol 3-kinase
PPAR: Peroxisome proliferator-activated receptor
PRRS: Pattern-recognition receptors
PTEN: Phosphatase and tensin homolog
RAB7: Member RAS oncogene family
RCT: Randomized controlled trial
RHOA: Ras homolog family member A
ROCK1: Rho associated coiled-coil containing protein kinase 1
ROS: Reactive oxygen species
SAEs: Serious adverse events
sEH: Soluble epoxide hydrolase
SF-36: Short-form 36
SIRT: Sirtuin
SOD: Superoxide dismutase
STAT3: Signal transducer and activator of transcription 3
TAP: Total adenylate pool
TCM: Traditional Chinese Medicine
TFAM: Transcription factor A, mitochondrial
TIE2: Tyrosine kinase with immunoglobin and epidermal growth factor homology domain 2
TLR: Toll-like receptor
TNF-α: Tumor necrosis factor-α
VCAM-1: Vascular cell adhesion molecule-1
VEGF: Vascular endothelial growth factor
VEGFR2: Vascular endothelial growth factor receptor 2
VPCs: Ventricular premature complexes
