# Supplementary figures and images for: Mechanisms and Efficacy of Traditional Chinese Medicine in Heart Failure
Source: Front Pharmacol. 2022 Feb 24;13:810587. doi: 10.3389/fphar.2022.810587 (PMC8908244; doi:10.3389/fphar.2022.810587)

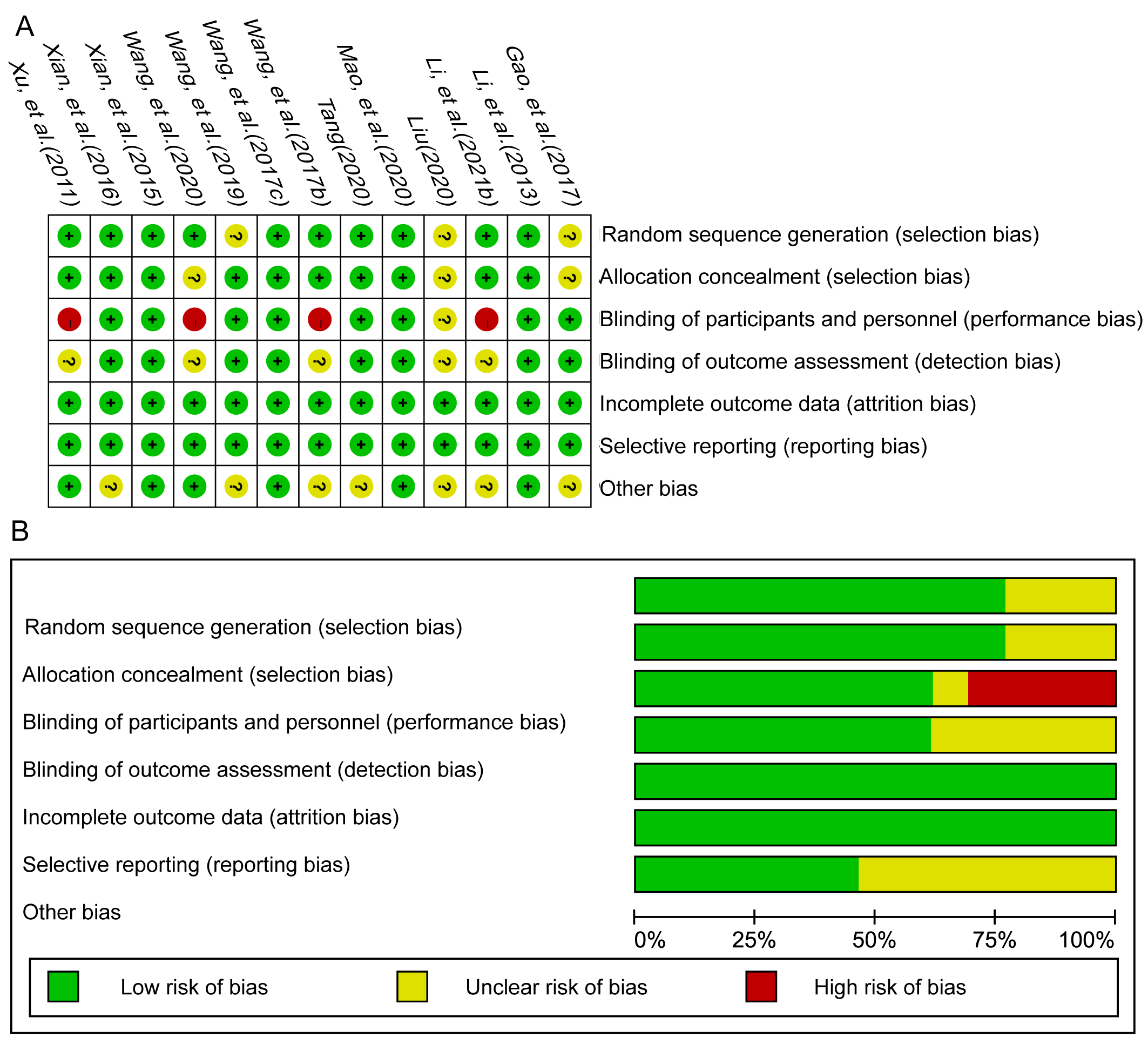

Supplement: Supplementary file 1 [file Image2.TIF]

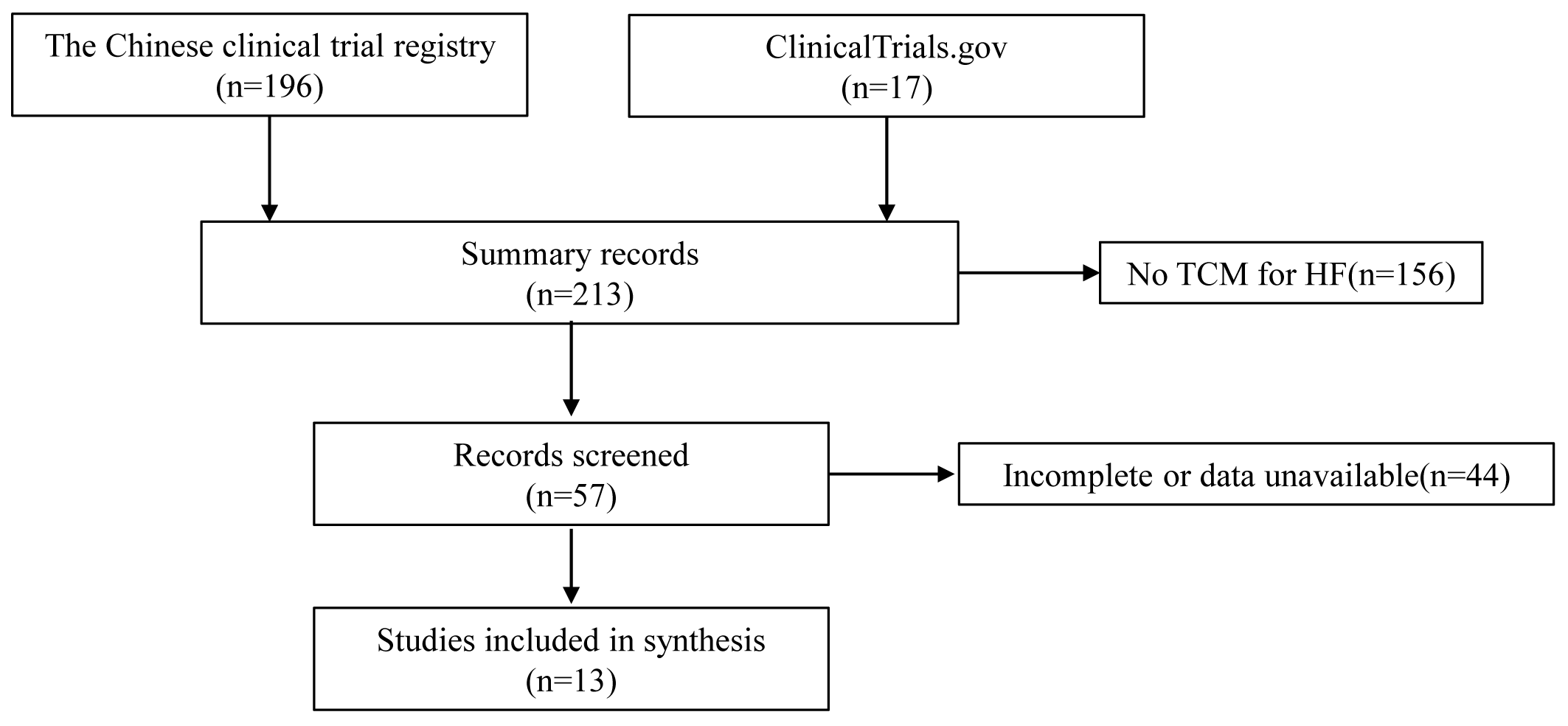

Supplement: Supplementary file 2 [file Image1.TIF]
